# New serological platform for detecting antibodies against *Mycobacterium tuberculosis* complex in European badgers

**DOI:** 10.1002/vms3.134

**Published:** 2019-01-18

**Authors:** Jose A. Infantes‐Lorenzo, Dipesh Dave, Immaculada Moreno, Paul Anderson, Sandrine Lesellier, Eamonn Gormley, Lucas Dominguez, Ana Balseiro, Christian Gortázar, Mercedes Dominguez, Francisco J. Salguero

**Affiliations:** ^1^ VISAVET Health Surveillance Centre Universidad Complutense de Madrid Madrid Spain; ^2^ Bacteriology Department Animal and Plant Health Agency Addlestone Surrey UK; ^3^ Unidad de Inmunología Microbiana Centro Nacional de Microbiología Instituto de Salud Carlos III Majadahonda Madrid Spain; ^4^ School of Veterinary Medicine University College Dublin (UCD) Dublin Ireland; ^5^ Departamento de Sanidad Animal Facultad de Veterinaria Universidad Complutense de Madrid Madrid Spain; ^6^ Centro de Biotecnología Animal Servicio Regional de Investigación y Desarrollo Agroalimentario (SERIDA) Deva‐Gijón Asturias Spain; ^7^ SaBio Instituto de Investigación en Recursos Cinegéticos IREC (CSIC‐UCLM‐JCCM) Ciudad Real Spain; ^8^ Department of Pathology and Infectious Diseases School of Veterinary Medicine University of Surrey Guildford UK

**Keywords:** Badgers, diagnosis, P22 ELISA, tuberculosis, wildlife reservoir

## Abstract

European badgers (*Meles meles*) have been identified as wildlife reservoirs for *Mycobacterium bovis* in the UK and Ireland, and may also have a role in the epidemiology of animal tuberculosis in other European regions. Thus, detection of *M. bovis*‐infected badgers may be required for the purposes of surveillance and monitoring of disease levels in infected populations. Current serological assays to detect *M. bovis* infection in live badgers, while rapid and inexpensive, show limited diagnostic sensitivity. Here we describe and evaluate new ELISA platforms for the recognition of the P22 multiprotein complex derived from the purified protein derivative (PPD) of *M. bovis*. The recognition of IgG against P22 multiprotein complex derived from PPD‐B was tested by ELISA in the serum of badgers from the UK, Ireland and Spain. TB infection in the badgers was indicated by the presence of *M. bovis* in tissues by culture and histology at post‐mortem examination and TB‐free status was established by repeated negativity in the interferon *γ* release assay (IGRA). In experimentally infected badgers, humoral antibody responses against P22 developed within 45 days post‐infection. The ELISA tests showed estimated sensitivity levels of 74–82% in experimentally and naturally infected badgers with specificities ranging from 75% to 100% depending on the badger population tested. The P22 multi‐antigen based ELISAs provide a sensitive and specific test platform for improved tuberculosis surveillance in badgers.

## Introduction

Tuberculosis caused by *M. bovis* and closely related members of the *Mycobacterium tuberculosis* complex remains one of the most important infectious diseases in cattle. Tuberculosis in cattle is controlled in Europe through routine test and slaughter policies. However, eradication of the disease can be hindered by the persistence of infection in wild animal reservoirs (Corner 2006) and European badgers (*Meles meles*) play an important role in the transmission cycle of bovine tuberculosis in the UK and Ireland (Donnelly *et al*. 2007; More 2009; Murphy *et al*. 2010) and possibly in other European regions (Balseiro *et al*. 2013; Bouchez‐Zacria *et al*. 2017).

The most sensitive tests for diagnosing tuberculosis in live badgers are currently based on cell‐mediated responses (Dalley *et al*. 2008; Balseiro *et al*. 2011). These tests can be difficult to implement on a large scale because they (1) require sufficient volumes of fresh blood, which need to be processed within a few hours after collection; (2) require more sophisticated expertise and laboratory equipment than ELISAs; (3) are more expensive than ELISAs; (4) require longer processing time and (5) cannot be used with *post‐mortem* samples. Antibody detection tests, in contrast, can be used to screen large numbers of animals because they are simple to perform on fresh and stored samples, require small sample volume, are relatively inexpensive and can be completed within 24 h. They can be performed and interpreted in parallel with cellular immune assays to increase overall sensitivity across the full spectrum of disease including when cellular anergy occurs (Green *et al*. 2009; Whelan *et al*. 2010; Chambers *et al*. 2011; Chambers 2013; Waters *et al*. 2015; Casal *et al*. 2017). However, the serological tests currently available for diagnosing TB in badgers, the DPP^®^ VetTB (lateral‐flow antibody detection test by Chembio, NY, USA) (Lyashchenko *et al*. 2013; Schaftenaar *et al*. 2013; Che’ *et al*. 2015; Vogelnest *et al*. 2015) that has recently replaced the BrockTB STAT‐PAK (Chembio, NY, USA) and a chemiluminescent multiplex immunoassay from Enfer Scientific (Co. Kildare, Ireland) (Aznar *et al*. 2017) are relatively insensitive: approximately 55% (S. Lesellier, personal communication) and 25.3% (Aznar *et al*. 2017) respectively in naturally infected badgers, depending on the cut‐off values applied. Both assays primarily target MPB83, MPB70, ESAT‐6 and CFP‐10.

In order to provide an expanded range of tests for large‐scale testing of *M. tuberculosis* complex infection in badgers, we have developed ELISA systems targeting antibodies against the recently described P22 multiprotein antigen (Infantes‐Lorenzo *et al*. 2017), which is affinity‐purified from bovine purified protein derivative (PPD) of *M. bovis* and has been shown to provide greater diagnostic sensitivity than conventional ELISAs in other host species in a high prevalence setting (Casal *et al*. 2017). In this study the P22‐based ELISAs were evaluated using serum samples from badgers infected naturally and experimentally with *M. bovis* and uninfected captive and wild badgers from three different European locations.

## Material and methods

### Serum sample collection

All serum samples were originally obtained by jugular venipuncture of anaesthetised badgers into serum separation vacutainer tubes (SST, Becton Dickinson™, New Jersey, USA). The general anaesthesia of the badgers was induced by intramuscular injection of ketamine hydrochloride (approximately 10 mg/kg, Vetalar, Boehringer Ingelheim) and medetomidine hydrochloride (approximately 0.1 mg/kg, Domitor, Pfizer) in Ireland, supplemented with butorphanol (approximately 0.1 mg/kg, Torbugesic R, ZoetisUKLtd, Tadworth, Surrey, UK) in the UK and Spain.

### Serum samples from uninfected and experimentally infected badgers from the UK

In the UK, a total of 36 captive badgers were infected with an average of 3 × 10^3^ CFU/mL viable *M. bovis* (spoligotype 9, strain 74/0449/97) by endobronchial instillation as described previously (Lesellier *et al*. 2011). Serum samples were obtained before the start of the study and at 0 (pre‐challenge), 15, 30, 45, 60 and 75 days post‐infection (dpi) (Table 1). All animals presented with TB visible lesions 75 days post‐infection (dpi) and *M. bovis* was cultured from the animal tissues (S. Lesellier, in preparation).

**Table 1:** Number, origin and characteristics of badger serum samples in this study

Country	n Animals	Sampling events	Positive animals	Negative animals	Infection pathway
United Kingdom	36	7*	34 *	36*	Experimental (bronchial)
Ireland	53	1	25†	28‡	Natural
Spain	32	1	–	32§	–

* Negative samples were obtained at pre‐challenge and 0 days post‐infection; positive samples were obtained at 15, 30, 45, 60 and 75 days post‐infection, two animals were removed from the dataset as blood samples were not obtained on all time‐points.

† Based on *M. bovis* culture and/or presence of visible lesions.

‡ Based on negative results in the *M. bovis* culture test and absence of visible and/or histological lesions.

§ Badgers were trapped in an area of low tuberculosis prevalence, and they tested negative for interferon gamma release.

### Serum samples from naturally infected and uninfected badgers from Ireland

Serum samples were originally collected from 53 badgers trapped at the end of a vaccine field study in Ireland (Gormley *et al*. 2017), and examined at *post‐mortem*. Animals were classified as tuberculosis‐positive (n = 25), if lesioned tissue sections showed the presence of acid‐fast bacilli based on Ziehl‐Neelsen staining or if at least one tissue gave a positive result in the *M. bovis* culture test. The remaining animals (n = 28) were classified as tuberculosis‐negative (Table 1).

### Serum samples from uninfected badgers from Spain

Thirty‐two badgers were captured in Asturias and tested negative for badger interferon gamma release assay (Table 1). These findings, together with the low local prevalence of tuberculosis in cattle and badgers (6.6% based on bacteriological culture from road‐killed badgers and 0.21% in cattle, Balseiro *et al*. 2011), allowed us to classify these animals as tuberculosis‐negative.

### Indirect P22 ELISA

An indirect ELISA was developed to detect antibodies against the *M*. *tuberculosis* complex P22 complex. This complex was affinity‐purified from bovine PPD [CZ Veterinaria (Porriño, Spain)] using a patented process (European Patent EP16382579) (Infantes‐Lorenzo *et al*. 2017). The ELISA was developed using previously described methods (Casal *et al*. 2017), with minor modifications. Maxisorp plates were coated with 10 *μ*g/mL P22 overnight at 4°C in phosphate‐buffered saline (PBS), then blocked with 5% skimmed milk powder solution (Central Lechera Asturiana, Spain) in PBS for 1 h at room temperature. After three washes with PBS containing 0.05% Tween‐20 (PBST), sera (100 *μ*L) were added to duplicate wells at 1:100 dilution in PBS‐skim milk and incubated for 60 min at 37°C. Horseradish peroxidase‐conjugated CF2/HRPo anti‐Badger IgG (100 *μ*L) (Goodger *et al*. 1994) was diluted to 1.5 *μ*g/mL in PBS and added to the plates, which were incubated for 30 min at room temperature. Then plates were incubated with 3,3′,5,5′‐tetramethylbenzidine substrate (Perbio) for 15 min in the dark at room temperature. The reaction was stopped by adding 100 *μ*L of 2 mmol/L H_2_SO_4_. Optical density was measured at 450 nm using an ELISA reader.

Negative control serum samples from UK tuberculosis‐free captive badgers were included in every plate in quadruplicate. Positive controls were obtained from UK badgers experimentally infected with *M. bovis*. Sample results were expressed as an ELISA percentage E%, calculated using the following formula (Che’ *et al*. 2015):

E%=mean sample optical density/(2×mean of negative control optical density)×100%

Cut‐off points were set‐up to calculate sensitivity and specificity: *E*% > 100, *E*% > 120 and *E*% > 150.

### Competitive P22 ELISA

As environmental mycobacteria can potentially interfere with tuberculosis diagnostic tests, we developed a competitive ELISA against P22 with the objective to exclude interference from cross‐reactive antibodies recognising antigens from environmental mycobacteria. This assay was developed and evaluated in the same way as the indirect ELISA, except that sera were diluted in skimmed milk supplemented with avian PPD at 150 *μ*g/mL (Casal *et al*. 2017). Samples were always assayed in parallel using the indirect and competitive ELISAs.

### Statistical analysis

Data were analysed using SPSS 20.0 (IBM, Somers, NY, USA). A Wilson 95% confidence interval (95%CI) was calculated for each percentage.

## Results

### Experimentally infected badgers from UK

**Figure 1:**
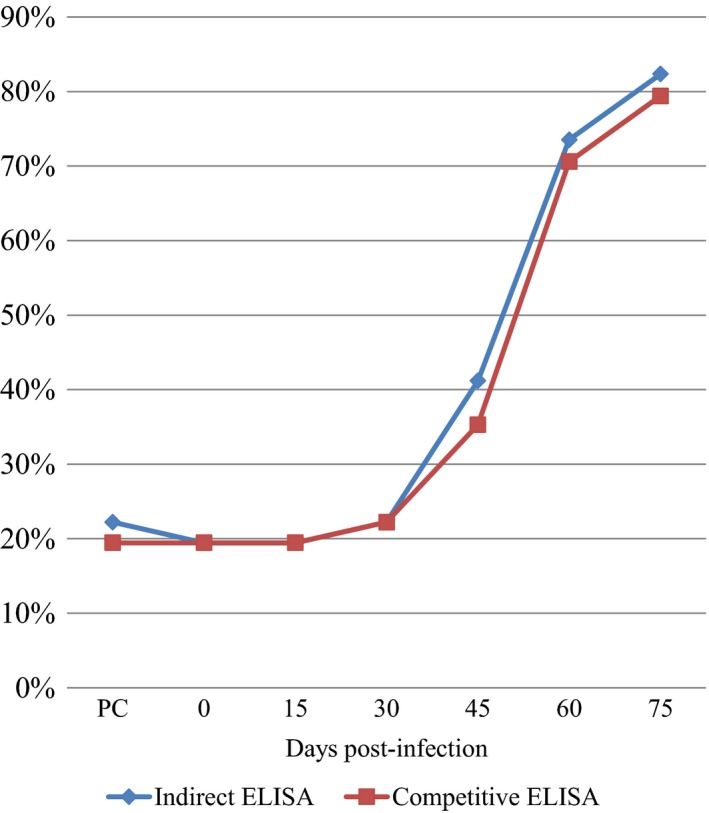
Proportion (%) of positive animals detected using indirect or competitive P22‐based ELISA at different time points after infection. PC = pre‐challenge time point.

The proportion of sero‐positive animals increased with time post‐infection in the indirect and competitive ELISAs (Fig. 1). Before challenge (pre‐challenge and T0), seven of the 36 badgers (19.44%) tested positive by indirect ELISA. One additional animal was only positive on one of these occasions and was not included in results shown in Table 2. As these animals were confirmed tuberculosis‐free at the time of testing, this pre‐challenge positivity was attributed to cross‐reactivity with other antigens similar to P22, possibly antigens secreted by *M*. *avium* or other environmental mycobacteria. Only one of the seven badgers that tested positive in the indirect ELISA tested negative in the competitive ELISA.

**Table 2:** Proportion (%) of badgers testing positive in an indirect (ID) or competitive (CP) ELISA detecting serum antibodies against the *Mycobacterium tuberculosis* complex (P22 protein complex) in experimental (United Kingdom) and natural infected badgers

		n Animals	Cut‐off 100%		Cut‐off 120%		Cut‐off 150%	
			ID	CP	ID	CP	ID	CP
TB infected	UK	34	82 (66–92)	79 (63–90)	73.53 (57–85)	73.53 (57–85)	64.71 (48–79)	64.71 (48–79)
	Ireland*	25	80 (61–91)	76 (57–89)	76 (57–89)	76 (57–89)	72 (52–86)	72 (53–86)
TB free	UK	36	19.44 (10–35)	16.66 (8–32)	19.44 (10–35)	16.66 (8–32)	19.44 (10–35)	16.66 (8–32)
	Ireland†	28	25 (13–43)	14.29 (1–27)	14.29 (6–31)	10.71 (4–27)	14.29 (6–31)	10.71 (4–27)
	Spain	32	3.13 (1–16)	0 (0–11)	0 (0–11)	0 (0–11)	0 (0–11)	0 (0–11)

Intervals in parentheses indicate 95% confidence limits.

* Based on *M. bovis* culture test.

† No visible lesions and culture‐negative.

The indirect and competitive ELISAs showed similar sensitivity of detection (82% and 79% respectively) of infected animals at the end of the study, day 75 (Table 2). At 60 dpi, 73.52% were found positive by the indirect ELISA (Fig. 1). As infection progressed, the mean E% increased from 127.30% before challenge to 336.26% at 75 dpi (Kruskal‐Wallis test, d.f. = 6, *P* < 0.001). The largest increase in the proportion of positive animals occurred approximately 1 month after infection, when the level of circulating antibodies was expected to rise. The rate of positive test responses for the period from 0 to 30 dpi differed significantly from the rate for the period from 45 to 75 dpi (Wilcoxon signed‐rank test, *P* < 0.001).

### Naturally infected and uninfected badgers from Ireland

Of 25 naturally infected badgers, 20 tested positive by the indirect ELISA and 19 by the competitive ELISA for the cut‐off point of 100%, yielding mean sensitivities of 80% and 76% respectively. The highest values tended to occur in animals with visible lesions (data not shown). The specificity of the competitive and indirect ELISAs was 85.71% when a cut‐off of *E*% = 100% was used, and increased to 89.29% with a cut‐off of 120%, but sensitivity decreased slightly (Table 2). The specificity was marginally lower in the indirect ELISA than in the competitive ELISA with both cut‐offs (75% and 85% respectively) (Table 2).

**Figure 2:**
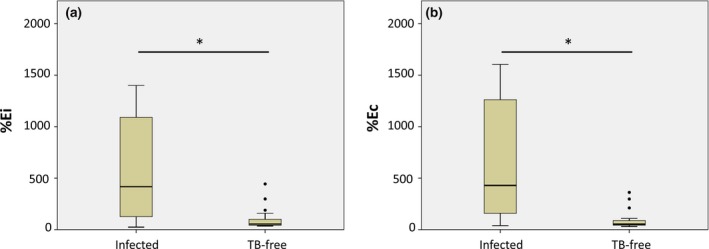
ELISA percentage (E%, see Methods) calculated based on the indirect ELISA (a) and competitive ELISA (b) with samples from infected and uninfected badgers from Ireland. Boxes indicate the lower and upper quartiles as well as median, and outliers are represented by closed circles. TB, tuberculosis. *Significant difference.

Figure 2 presents the median levels of serological responses, which differed significantly between naturally infected and uninfected badgers (Mann–Whitney, *P* < 0.001).

### Uninfected badgers from Spain

Of 32 tuberculosis‐free Spanish badgers, only one tested positive in the indirect ELISA, giving a specificity of 96.88%. No animal tested positive in the competitive ELISA, giving a 100% specificity (Table 2).

## Discussion

Our results suggest that the indirect and competitive P22 ELISA tests described here have the potential to provide greater levels of sensitivity compared to other anti‐*M. tuberculosis* complex antibody detection tests currently used in badgers. These inexpensive and straightforward tests require small serum volumes and their application could help improve tuberculosis diagnostics and field studies in badgers.

In experimentally infected badgers, we obtained high sensitivities ranging from 73.52% at 60 dpi to 82.35% at 75 dpi. When applied to samples from badgers from Ireland, the indirect ELISA showed sensitivity levels of 80% and the competitive ELISA showed 76% sensitivity. These are considerably higher than the sensitivities of 25.3–55% reported for other antibody tests for badgers (Clifton‐Hadley *et al*. 1995; Greenwald *et al*. 2003; Chambers *et al*. 2010; Aznar *et al*. 2017). They are also consistent with high sensitivity observed in infected cattle (Casal *et al*. 2017). From a disease management point of view, the test would likely be applied in populations with high infection levels, using high levels of sensitivity in order to maximise the detection of infected animals.

The specificity of the serological assays varied between badger populations/countries of origin, possibly as the result of potential exposure to different environment mycobacterial species. It reached 100% in a Spanish badger captive population and 90% in uninfected badgers from the Republic of Ireland when using the cut‐off point of *E* = 120% in the competitive ELISA. However, lower specificity results were recorded when we used samples from apparent tuberculosis‐free badgers from the UK. This is suggestive of different levels of immune sensitisation in the different countries: *M. avium* infection in badgers has previously been reported (Balseiro *et al*. 2011), In addition, we cannot rule out that all of the UK badgers were genuinely TB free. The different specificity levels for the same interpretation criteria suggest that the UK badgers used in this study may have been previously exposed to cross‐reactive antigens homologous to P22.

The competitive ELISA using avian PPD antigen did not resolve the relatively low specificity measured with samples from UK badgers. Although P22 is composed mainly of MPB70 and MPB83, it shares several proteins with the *M*. *avium* complex that may contribute to cross‐reactivity (Infantes‐Lorenzo *et al*. 2017). In addition, MPB70 and MPB83 show high sequence similarity to proteins from nontuberculous mycobacteria that do not form part of the *M. avium* complex, such as proteins from *M. kansasii* (77% identity, 98% coverage) and *M. abscessus* (73% identity, 82% coverage) (www.uniprot.org, accessed 17 November 2017). For screening tests specificity is particularly important in areas of low tuberculosis prevalence, as it minimises the disclosure of false‐positive test results. However, a potential problem with determining overall specificity in different environments is that the causative cross‐reactive agent is often unknown, and it is likely to vary between regions. The most appropriate way to address this is to modify the cut‐off points to take into account the many confounders in local environments. Specificity can be improved by adjusting the cut‐off value, albeit potentially at the expense of sensitivity. With a cut‐off value of 100%, the indirect ELISA showed specificity levels of 75% and the competitive ELISA showed a specificity of 85.71%. Increasing the cut‐off to 120% increased specificity of the indirect assay to 85.71% and specificity of the competitive assay to 89.29%, while sensitivity remained stable at 76%.

It is possible that the true specificity of our assays is even higher than we measured, because bacterial culture is not a gold standard test, even when performed on necropsy tissues (Crawshaw *et al*. 2008), Thus, it is possible that some badgers were infected with members of *M. tuberculosis* complex but were not detected *post‐mortem* based on mycobacterial culture or pathology. Molecular characterisation of *M. tuberculosis* complex strains isolated from animals in the UK has revealed the presence of *M. microti*, and a small proportion of the spoligotypes was associated with badgers (Smith *et al*. 2009). Although we have no evidence of *M. microti* infections in any of the badgers used in this study, we cannot rule out the possibility that any such infection could potentially impact on the specificity of the test. Despite our ELISAs disclosing positive results for 20 of 25 culture‐positive, naturally infected badgers, some of the culture‐negative animals may have had subclinical infection; this would affect the reliability of our measured sensitivity and specificity. The results from the different countries highlight that test performance can vary according to different environments and cut‐off point needs to be established and adapted to local conditions. Nevertheless, our high specificity observed with samples from uninfected badgers from Spain suggests that both ELISAs perform well.

The rates of test positive results in each ELISA increased with disease progression. This indicates an increase in sensitivity associated with more advanced infection, as reported in experimentally infected wild boar (*Sus scrofa*) and experimentally infected red deer (*Cervus elaphus*) (Garrido *et al*. 2011; Thomas *et al*. 2017). In badgers, the sensitivity of the BrockTB STAT‐PAK appeared to be higher in animals with more severe tuberculosis; those with lesions visible at *post‐mortem* (Clifton‐Hadley *et al*. 1995; Chambers *et al*. 2008). This ability to detect animals with advanced disease is particularly important because they are potentially the principal shedders of *M. bovis*, and may pose a higher risk of bacterial transmission to livestock (Gallagher *et al*. 1998; Delahay *et al*. 2013).

## Conclusion

In conclusion, the development of novel serological tests based on the P22 multiprotein antigen may lead to more sensitive and specific ELISAs, and prove to be an effective tool to support the detection and management of tuberculosis in badgers.

## Source of funding

This work was supported by the Instituto Nacional de Investigación y Tecnología Agraria y Alimentaria of Spain (INIA; RTA2015‐00043‐C02‐02) and the TAVS‐CM Programme of the Comunidad de Madrid (S2013/ABI‐2747), cofinanced by the FEDER fund ‘A way to build Europe’. This work was partially supported by a FEDER co‐funded grant from INIA (RTA2014‐00002‐C02‐01). Jose Antonio Infantes‐Lorenzo was supported by an FPU contract‐fellowship (Formación de Profesorado Universitario) from the Ministerio de Educación, Cultura y Deporte of the Spanish Government (FPU2013/6000).

## Conflicts of interest

The authors declare that they have no conflicts of interest.

## Ethics statement

All animal procedures conducted for this study were approved by the Animal Welfare and Ethical Review Board at the UK Animal and Plant Health Agency (APHA) [under the Animal (Scientific Procedures) Act 1986 and Home Office authorized Project License 70/7878], the University College Dublin Animal Research Ethics Committee (AREC‐P‐08‐26), and research licence (B100/3187) issued by the Dept of Health & Children, Ireland, and the Animal Research Ethics Committee of the Principado de Asturias, Spain (PROAE 20‐2015).

## Contributions

JIL and DD performed the experiments, analyzed and interpreted the data and wrote the first draft of the manuscript. JIL, SL, EG and JS were involved in interpreting the data. DD, PA, SL, AB and EG obtained serum samples from animals. CG and LD analyzed the data and revised the manuscript. JIL, JS, SL and MD designed the study, analyzed the data, and revised the manuscript. IM, SL, EG, LD, AB and CG critically revised the manuscript. All authors have read and approved the final manuscript.

## References

[vms3134-bib-0001] Aznar I. , Frankena K. , More S.J. , O'Keeffe J. , McGrath G. & de Jong M.C.M. (2017) Quantification of *Mycobacterium bovis* transmission in a badger vaccine field trial. Preventive Veterinary Medicine 149, 29–37. 29290298 10.1016/j.prevetmed.2017.10.010

[vms3134-bib-0002] Balseiro A. , Rodriguez O. , Gonzalez‐Quiros P. , Merediz I. , Sevilla I.A. , Dave D. *et al* (2011) Infection of Eurasian badgers (*Meles meles*) with *Mycobacterium bovis* and *Mycobacterium avium* complex in Spain. The Veterinary Journal 190, e21–e25. 21612958 10.1016/j.tvjl.2011.04.012

[vms3134-bib-0003] Balseiro A. , González‐Quirós P. , Rodríguez Ó. , Copano M.F. , Merediz I. , de Juan L. *et al* (2013) Spatial relationships between Eurasian badgers (*Meles meles*) and cattle infected with *Mycobacterium bovis* in Northern Spain. The Veterinary Journal 197, 739–745. 23602422 10.1016/j.tvjl.2013.03.017

[vms3134-bib-0004] Bouchez‐Zacria M. , Courcoul A. , Jabert P. , Richomme C. & Durand B. (2017) Environmental determinants of the *Mycobacterium bovis* concomitant infection in cattle and badgers in France. European Journal of Wildlife Research 63, 74.

[vms3134-bib-0005] Casal C. , Infantes J.A. , Risalde M.A. , Díez‐Guerrier A. , Domínguez M. , Moreno I. *et al* (2017) Antibody detection tests improve the sensitivity of tuberculosis diagnosis in cattle. Research in Veterinary Science 112, 214–221. 28521256 10.1016/j.rvsc.2017.05.012

[vms3134-bib-0006] Chambers M.A. (2013) Review of the diagnosis of tuberculosis in non‐bovid wildlife species using immunological methods–an update of published work since 2009. Transboundary and Emerging Diseases 60(Suppl 1), 14–27. 10.1111/tbed.12094 24171845

[vms3134-bib-0007] Chambers M.A. , Crawshaw T. , Waterhouse S. , Delahay R. , Hewinson R.G. & Lyashchenko K.P. (2008) Validation of the BrockTB stat‐pak assay for detection of tuberculosis in Eurasian badgers (*Meles meles*) and influence of disease severity on diagnostic accuracy. Journal of Clinical Microbiology 46, 1498–1500. 18272706 10.1128/JCM.02117-07 PMC2292970

[vms3134-bib-0008] Chambers M.A. , Lyashchenko K.P. , Greenwald R. , Esfandiari J. , James E. , Barker L. *et al* (2010) Evaluation of a rapid serological test for the determination of *Mycobacterium bovis* infection in badgers (*Meles meles*) found dead. Clinical and Vaccine Immunology 17, 408–411. 20042520 10.1128/CVI.00424-09 PMC2837968

[vms3134-bib-0009] Chambers M.A. , Rogers F. , Delahay R.J. , Lesellier S. , Ashford R. , Dalley D. *et al* (2011) Bacillus Calmette‐Guerin vaccination reduces the severity and progression of tuberculosis in badgers. Proceedings of the Royal Society B: Biological Sciences 278, 1913–1920. 10.1098/rspb.2010.1953 PMC3097825 21123260

[vms3134-bib-0010] Che’ A.A. , Gonzalez‐Barrio D. , Ortiz J.A. , Diez‐Delgado I. , Boadella M. , Barasona J.A. *et al* (2015) Testing Eurasian wild boar piglets for serum antibodies against *Mycobacterium bovis* . Preventive Veterinary Medicine 121, 93–98. 26051843 10.1016/j.prevetmed.2015.05.011

[vms3134-bib-0011] Clifton‐Hadley R.S. , Sayers A.R. & Stock M.P. (1995) Evaluation of an ELISA for *Mycobacterium bovis* infection in badgers (*Meles meles*). Veterinary Record 137, 555–558. 8644433 10.1136/vr.137.22.555

[vms3134-bib-0012] Corner L.A. (2006) The role of wild animal populations in the epidemiology of tuberculosis in domestic animals: how to assess the risk. Veterinary Microbiology 112, 303–312. 16326039 10.1016/j.vetmic.2005.11.015

[vms3134-bib-0013] Crawshaw T.R. , Griffiths I.B. & Clifton‐Hadley R.S. (2008) Comparison of a standard and a detailed postmortem protocol for detecting *Mycobacterium bovis* in badgers. Veterinary Record 163, 473–477. 18931354 10.1136/vr.163.16.473

[vms3134-bib-0014] Dalley D. , Dave D. , Lesellier S. , Palmer S. , Crawshaw T. , Hewinson R.G. & Chambers M. (2008) Development and evaluation of a gamma‐interferon assay for tuberculosis in badgers (*Meles meles*). Tuberculosis 88, 235–243. 18083067 10.1016/j.tube.2007.11.001

[vms3134-bib-0015] Delahay R.J. , Walker N. , Smith G.C. , Wilkinson D. , Clifton‐Hadley R.S. , Cheeseman C.L. *et al* (2013) Long‐term temporal trends and estimated transmission rates for *Mycobacterium bovis* infection in an undisturbed high‐density badger (*Meles meles*) population. Epidemiology and Infection 141, 1445–1456. 23537573 10.1017/S0950268813000721 PMC9151602

[vms3134-bib-0016] Donnelly C.A. , Wei G. , Johnston W.T. , Cox D.R. , Woodroffe R. , Bourne F.J. *et al* (2007) Impacts of widespread badger culling on cattle tuberculosis: concluding analyses from a large‐scale field trial. International Journal of Infectious Diseases 11, 300–308. 17566777 10.1016/j.ijid.2007.04.001

[vms3134-bib-0017] Gallagher J. , Monies R. , Gavier‐Widen M. & Rule B. (1998) Role of infected, non‐diseased badgers in the pathogenesis of tuberculosis in the badger. Veterinary Record 142, 710–714. 9682428 10.1136/vr.142.26.710

[vms3134-bib-0018] Garrido J.M. , Sevilla I.A. , Beltran‐Beck B. , Minguijon E. , Ballesteros C. , Galindo R.C. *et al* (2011) Protection against tuberculosis in Eurasian wild boar vaccinated with heat‐inactivated *Mycobacterium bovis* . PLoS ONE 6, e24905. 21935486 10.1371/journal.pone.0024905 PMC3173485

[vms3134-bib-0019] Goodger J. , Russell W.P. , Nolan A. & Newell D.G. (1994) Production and characterization of a monoclonal badger anti‐immunoglobulin G and its use in defining the specificity of *Mycobacterium bovis* infection in badgers by western blot. Veterinary Immunology and Immunopathology 40, 243–252. 8160362 10.1016/0165-2427(94)90023-x

[vms3134-bib-0020] Gormley E. , Bhuachalla D.N. , O'Keeffe J. , Murphy D. , Aldwell F.E. , Fitzsimons T. *et al* (2017) Oral vaccination of free‐living badgers (*Meles meles*) with Bacille Calmette Guerin (BCG) vaccine confers protection against tuberculosis. PLoS ONE 12, e0168851. 28121981 10.1371/journal.pone.0168851 PMC5266210

[vms3134-bib-0021] Green L.R. , Jones C.C. , Sherwood A.L. , Garkavi I.V. , Cangelosi G.A. , Thacker T.C. *et al* (2009) Single‐antigen serological testing for bovine tuberculosis. Clinical and Vaccine Immunology 16, 1309–1313. 19605596 10.1128/CVI.00028-09 PMC2745019

[vms3134-bib-0022] Greenwald R. , Esfandiari J. , Lesellier S. , Houghton R. , Pollock J. , Aagaard C. *et al* (2003) Improved serodetection of *Mycobacterium bovis* infection in badgers (*Meles meles*) using multiantigen test formats. Diagnostic Microbiology and Infectious Disease 46, 197–203. 12867095 10.1016/s0732-8893(03)00046-4

[vms3134-bib-0023] Infantes‐Lorenzo J.A. , Moreno I. , de los Ángeles Risalde M. , Roy Á. , Villar M. , Romero B. *et al* (2017) Proteomic characterisation of bovine and avian purified protein derivatives and identification of specific antigens for serodiagnosis of bovine tuberculosis. Clinical Proteomics 14, 36. 29142508 10.1186/s12014-017-9171-z PMC5669029

[vms3134-bib-0024] Lesellier S. , Palmer S. , Gowtage‐Sequiera S. , Ashford R. , Dalley D. , Dave D. *et al* (2011) Protection of Eurasian badgers (*Meles meles*) from tuberculosis after intra‐muscular vaccination with different doses of BCG. Vaccine 29, 3782–3790. 21440035 10.1016/j.vaccine.2011.03.028

[vms3134-bib-0025] Lyashchenko K.P. , Greenwald R. , Esfandiari J. , O'Brien D.J. , Schmitt S.M. , Palmer M.V. & Waters W.R. (2013) Rapid detection of serum antibody by dual‐path platform VetTB assay in white‐tailed deer infected with *Mycobacterium bovis* . Clinical and Vaccine Immunology 20, 907–911. 23595504 10.1128/CVI.00120-13 PMC3675973

[vms3134-bib-0026] More S.J. (2009) What is needed to eradicate bovine tuberculosis successfully: an Ireland perspective. The Veterinary Journal 180, 275–278. 19289233 10.1016/j.tvjl.2009.01.027

[vms3134-bib-0028] Murphy D. , Gormley E. , Costello E. , O'Meara D. & Corner L.A. (2010) The prevalence and distribution of *Mycobacterium bovis* infection in European badgers (*Meles meles*) as determined by enhanced post mortem examination and bacteriological culture. Research in Veterinary Science 88, 1–5. 19545882 10.1016/j.rvsc.2009.05.020

[vms3134-bib-0029] Schaftenaar W. , Lecu A. , Greenwald R. & Lyashchenko K.P. (2013) Retrospective serological investigation of bovine tuberculosis in two gemsbok (Oryx gazelle gazelle) and an onager (Equus hemionus onager). Journal of Zoo and Wildlife Medicine 44, 1036–1042. 24450065 10.1638/2013-0064R.1

[vms3134-bib-0030] Smith N.H. , Crawshaw T. , Parry J. & Birtles R.J. (2009) Mycobacterium microti: more diverse than previously thought. Journal of Clinical Microbiology 47, 2551–2559. 19535520 10.1128/JCM.00638-09 PMC2725668

[vms3134-bib-0031] Thomas J. , Risalde M.A. , Serrano M. , Sevilla I. , Geijo M. , Ortiz J.A. *et al* (2017) The response of red deer to oral administration of heat‐inactivated *Mycobacterium bovis* and challenge with a field strain. Veterinary Microbiology 208, 195–202. 28888638 10.1016/j.vetmic.2017.08.007

[vms3134-bib-0032] Vogelnest L. , Hulst F. , Thompson P. , Lyashchenko K.P. & Herrin K.A. (2015) Diagnosis and management of tuberculosis (*Mycobacterium tuberculosis*) in an Asian elephant (*Elephas maximus*) with a newborn calf. Journal of Zoo and Wildlife Medicine 46, 77–85. 25831579 10.1638/2014-0024R1.1

[vms3134-bib-0033] Waters W.R. , Palmer M.V. , Stafne M.R. , Bass K.E. , Maggioli M.F. , Thacker T.C. *et al* (2015) Effects of serial skin testing with purified protein derivative on the level and quality of antibodies to complex and defined antigens in *Mycobacterium bovis*‐infected cattle. Clinical and Vaccine Immunology 22, 641–649. 25855555 10.1128/CVI.00119-15 PMC4446403

[vms3134-bib-0034] Whelan C. , Whelan A.O. , Shuralev E. , Kwok H.F. , Hewinson G. , Clarke J. & Vordermeier H.M. (2010) Performance of the Enferplex TB assay with cattle in Great Britain and assessment of its suitability as a test to distinguish infected and vaccinated animals. Clinical and Vaccine Immunology 17, 813–817. 20219883 10.1128/CVI.00489-09 PMC2863378

